# Effect of lamotrigine on seizure development in a rat pentylenetetrazole kindling model

**DOI:** 10.1002/brb3.727

**Published:** 2017-05-31

**Authors:** Yishu Chen, Xiaokuo He, Qianqian Sun, Ziyan Fang, Liemin Zhou

**Affiliations:** ^1^ Department of Neurology The First Affiliated Hospital Sun Yat‐Sen University Guangzhou Guangdong Province China; ^2^ Rehabilitation Medicine Center Taihe Hospital Shiyan Hubei Province China; ^3^ Department of Rehabilitation Medicine Fujian University of Traditional Chinese Medicine Fuzhou Fujian Province China; ^4^ Department of Neurology The Affiliated Brain Hospital of Guangzhou Medical University (Guangzhou Huiai Hospital) Guangzhou China

**Keywords:** antiepileptogenesis, kindling, lamotrigine, pentylenetetrazole, seizure

## Abstract

**Introduction:**

Epileptogenesis is a process of seizure development. Lamotrigine is a novel antiepileptic drug which is also used for antiepileptogenic research. Kindling models are recommended as potentially useful tools for antiepileptogenic treatment discovery. However, previous studies demonstrated that the antiepileptogenic effect of lamotrigine is controversial in the electrical kindling model. Chemical kindling such as with pentylenetetrazole is another kindling model. The aims of this study were to examine whether lamotrigine could prevent the development of seizure in pentylenetetrazole kindling rats.

**Methods:**

Female rats were kindled by subconvulsive doses of pentylenetetrazole (35 mg/kg) once every other day for 15 times. Thereafter, the kindled rats received different doses of lamotrigine (5, 10 and 20 mg/kg) before pentylenetetrazole to observe the anticonvulsant effect. For the antiepileptogenic experiment, rats were kindled as the same way while pretreated (1 h) with different doses of lamotrigine (5, 10 and 20 mg/kg) before each injection of pentylenetetrazole. After a washout period for 1 week, the rats were administrated with pentylenetetrazole again for 3 times. The seizures were recorded each time. Later it was in vivo electrophysiological experiments followed with histologic analysis.

**Results:**

For the anticonvulsant experiment lamotrigine dose‐dependently suppressed pentylenetetrazole‐induced seizures. Here, 20 mg/kg of lamotrigine pretreatment significantly blocked the seizure development in rats for their seizure stages remained longer in 1–3 during the kindling phase. Mean seizure stages or generalized seizure durations in the 10 and 20 mg/kg lamotrigine pretreated groups were significantly lower or shorter when received 3 times of pentylenetetrazole after the washout period. Electrophysiological study also demonstrated 20 mg/kg of lamotrigine pretreatment obviously eliminated increased population spike amplitude in hippocampus. However, different doses of lamotrigine pretreatment could not alleviate severity of hippocampal neuronal damage.

**Conclusions:**

The results suggest that adequate doses of lamotrigine can prevent seizure development in the pentylenetetrazole kindling rat model.

## 1 INTRODUCTION

Epilepsy is a chronic neurological disease and affects about 1% of the population (Berg et al., 2010; Loscher & Brandt, 2010). Besides recurrent seizures, people who suffer this disease may also have changes in their consciousness and motor abilities. However, about 30% of patients with epilepsy are resistant to current antiepileptic drugs (AEDs) (Chadwick, 1990; Pierzchala, 2010). Therefore, in addition to controlling seizures, there is a hope for drugs, especially AEDs can suppress epileptogenesis, a process of seizure development, which results from a variety of brain insults such as genetic abnormalities, stroke, infections, status epilepticus (SE) or others (Acharya, Hattiangady, & Shetty, 2008; Loscher & Brandt, 2010).

In recent decades, a few animal models of epileptogenesis have been developed, which have greatly strengthened our understanding of the processes leading to epilepsy and thus of methods for antiepileptogenic therapies (Diaz‐Arrastia, Agostini, Madden, & Van Ness, 2009; Stables et al., 2003). Kindling and poststatus epilepticus (SE) temporal lobe epilepsy (TLE) are two models that were recommended as potentially useful tools for antiepileptogenic treatment discovery (Stables et al., 2003). Kindling is referred to repetitive and intermittent administration of subconvulsant chemical or electrical stimulations can induce progressive amplification of seizures (Dhir, 2012). Fully kindled seizures resemble complex partial seizures with secondary generalization, so that kindling is considered as a model of TLE in humans (Loscher & Brandt, 2010; Stables et al., 2003). Evidence suggests that different kindling protocols result in different outcomes. As for electrical kindling models, of the current AEDs, previous experiments have proved that only valproate, phenobarbital, and levetiracetam, but not including lamotrigine (LTG) have the powerful antikindling effects (Loscher & Brandt, 2010). In their designs, animals were administered subconvulsant electrical stimulations with AEDs was followed by a washout phase and then stimulations were resumed in the absence of drugs, which can exclude the possibility that AEDs simply mask the expression of kindled seizures through an anticonvulsant action. However, this protocol needs long‐term implantation of an electrode into a region of the temporal lobe such as amygdala or hippocampus, which cannot rule out the interference of the brain injury caused by electrode implantation (Loscher, Wahnschaffe, Honack, & Rundfeldt, 1995; McIntyre, Poulter, & Gilby, 2002; Morimoto, Fahnestock, & Racine, 2004; Niespodziany, Klitgaard, & Margineanu, 1999). Chemical kindling models are administration of certain chemical agents systemically, such as pentylenetetrazole (PTZ), a GABAA receptor antagonist. These models are easier and less time consuming compared to electrical kindling, and do not require brain surgery or implantation of electrodes (Dhir, 2012). So chemical kindling may be another model of epileptogenesis available for antiepileptogenesis drug screening. However, to our knowledge, there were no direct antiepileptogenic experiments with AEDs in chemical kindling models (Acharya et al., 2008; Loscher & Brandt, 2010).

Lamotrigine is a novel broad‐spectrum AED widely used in the treatment of epilepsy such as partial, secondarily generalized, and tonic‐clonic seizures (Messenheimer, 1995; Moeller, Rahey, & Sadler, 2009; Warshavsky, Eilam, & Gilad, 2016). For electrical kindling, as far as we know there was a study to observe the antiepileptogenic activity of LTG with a dose of 20 mg/kg in rats, and it proved LTG can delay the development of kindling, but cannot further stop the kindling acquisition after a washout phase (Stratton, Large, Cox, Davies, & Hagan, 2003). Some other studies found LTG is either without effect (5 mg/kg) or even facilitating (15 mg/kg) the electrical kindling development, which may be related to different stimulation parameter settings (Krupp, Heynen, Li, Post, & Weiss, 2000; Postma, Krupp, Li, Post, & Weiss, 2000). For chemical kindling such as PTZ kindling, to our knowledge, there were two indirect studies that demonstrated rodent treatment of 5 mg/kg of LTG before each PTZ stimulation, which did not influence the development of kindling, but lead to tolerance to a higher dose of LTG and carbamazepine (Singh, Pillai, & Mehndiratta, 2014; Srivastava, Woodhead, & White, 2003). Post‐SE TLE models are recommended as potentially useful tools for antiepileptogenic treatment discovery (Stables et al., 2003). There is a latent period between SE and onset of spontaneous seizures for administration of drugs in these models. However, previous studies proved LTG has no determined antiepileptogenic effect in these models as well(Stables et al., 2003). In view of the insufficient or contradictory evidences about the antiepileptogenic role of LTG in the above animal models, this study was designed to examine whether LTG has an antiepileptogenic action in PTZ kindling model, in which LTG was administered to rats with different doses before PTZ stimulations.

Underlying pathologic and electrophysiological changes usually occur in parallel with seizures, for instance, neuronal damage is implicated in the development of seizure (Radzik, Miziak, Dudka, Chroscinska‐Krawczyk, & Czuczwar, 2015; Sutula, 2002), and neuronal hyperexcitability and paired‐pulse inhibition of neuronal activity are important electrophysiological features of epilepsy (Gorter, van Vliet, & Lopes da Silva, 2016). A number of experiments proved LTG has a neuroprotective effect when started during or soon after brain insults because of alleviating neuronal damage (Ketter, Manji, & Post, 2003; Malik, Arif, & Hirsch, 2006; Nissinen, Large, Stratton, & Pitkanen, 2004). It is believed that there is a clear‐cut relationship between neuroprotection and inhibition of epileptogenesis (Loscher & Brandt, 2010; Radzik et al., 2015).Thus, in addition to the behavioral research, electrophysiology and pathology were also observed to evaluate the effect of LTG on seizure development in our study.

## 2 MATERIALS AND METHODS

### 2.1 Animals

Female Sprague‐Dawley rats weighing 220–250 g were used. They were kept under controlled environmental conditions (ambient temperature 24–25°C, humidity 50–60%, 12/12 hr light/dark cycle). The animals had free access to food and water. All experiments were done at the same time in the morning to avoid the bias of circadian rhythms. The study was carried out in strict accordance with the Guide for the Care and Use of Laboratory Animals of the National Institutes of Health. The protocol was approved by the Animal Care Committee of Sun Yat‐sen University.

### 2.2 Drugs and reagents

LTG (Sigma Laboratories) was freshly suspended in 0.5% (W/V) methylcellulose (MC) before administration. PTZ (Sigma Laboratories) was freshly dissolved in saline. All drugs and vehicles were given in a volume of 1 ml/kg and delivered intraperitoneally (i.p.).

### 2.3 PTZ kindling

Kindling was according to the method described by Srivastava et al. (2003) and Dhir (2012). Rats were kindled by injections of a subconvulsant dose of PTZ (35 mg/kg) with or without LTG 1 hr before once every other day. Behaviors were observed for a period of 30 min. Seizure activity was scored, using the revised Racine's scale described by Racine as follows (Treiman, Walton, & Kendrick, 1990): Stage 0: No response, Stage 1: Ear and facial twitching, Stage 2: Myoclonic body jerks without upright position, Stage 3: Myoclonic jerks, upright position with clonic forelimb convulsions, Stage 4: Tonic‐clonic seizures, Stage 5: Generalized tonic‐clonic seizures, loss of postural control. Seizure duration was only measured when a generalized seizure, i.e., stage 4 or 5, occurred. Rats that had seizure stages of 4 or 5 after three consecutive injections of PTZ were defined as fully kindled (Davoudi, Shojaei, Palizvan, Javan, & Mirnajafi‐Zadeh, 2013).

### 2.4 Electrophysiological experiments

Rats were anesthetized with urethane (1.5 g/kg, i.p.) and placed into a stereotaxic apparatus. Body temperatures were maintained at 37°C. Holes were drilled in the skull, the recording electrode was directed toward the dentate gyrus (4 mm posterior to bregma, 2 mm lateral to the midline, depth of 3 mm ventral to brain surface), and the stimulation electrode was directed toward the ipsilateral perforant path (7 mm posterior to bregma, 4 mm lateral to the midline, depth of 3.5 mm ventral to brain surface). A stainless clip was clamped to the skin serving as a reference electrode. Two types of input–output curves were recorded in this study: (1) Population spike (PS) amplitude: Monophasic pulse (0.1 ms, 0.1 mA) was applied to evoke PS through a stimulating isolator, and when the amplitude reached half of the maximum, it was recorded; and (2) Paired‐pulse interaction (inhibition/facilitation): 10 min later, the response to paired‐pulse stimulation was recorded with an interstimulus interval at 20 ms and 90 ms. The PS amplitude was measured from the peak negativity of the spike to the point at which a vertically drawn line would intersect a line drawn between the two positive maxima of the response ((a + b) / 2), as showed in Figure 4a. The paired‐pulse index was a value that was calculated through the dividing amplitude of the second population spike (PS_2_) by the first population spike (PS_1_) which is expressed as PS_2_ / PS_1_, as showed in Figure 5a (He, Yan, Chen, & Ran, 2012; Waldbaum & Dudek, 2009).

### 2.5 Histologic analysis of brain tissue

Upon completion of the electrophysiological experiments, rats were deeply anesthetized and perfused intracardially. Their brains were removed, frozen, and cut into 10‐μm‐thick coronal sections and stained with cresyl violet. Histological analyses were performed to verify the correct location of electrodes in hippocampus and count number of surviving neurons per 1 mm length in CA1, CA3, and DG subfields of the hippocampus blindly with light microscopy. We counted neurons on each subfield in selected randomly every 10th section. Detailed procedure was carried out as described (Wang et al., 2008).

### 2.6 Experimental design

Due to lower mortality rates than males and LTG do not influence the sex hormone axis, female rats were used as experimental animals (Scharfman & MacLusky, 2014; Svalheim, Sveberg, Mochol, & Tauboll, 2015).There were two sets of experiments. For the anticonvulsant experiment, 30 rats were kindled by injections of a subconvulsant dose of PTZ (35 mg/kg) once every other day for 15 times. Our preliminary experiment found most rats were full kindled for 15 stimulations. Thereafter 1 week later, the kindled rats were randomly divided into 4 groups and the acute received vehicle or different doses of LTG (5, 10 and 20 mg/kg, respectively) 1 hr before 35 mg/kg of PTZ to test the anticonvulsant effect.

Another was the antiepileptogenic experiment. Rats were randomly divided into 5 groups (*n *= 10 per group): control group (vehicle + vehicle), vehicle‐treated group (vehicle + PTZ) and three LTG‐treated groups (LTG + PTZ). First rats were kindled by injections of PTZ (35 mg/kg) with LTG or vehicle 1 hr before once every other day 15 times and their behavior was observed. In the three LTG‐treated groups, the doses of LTG were 5, 10, and 20 mg/kg, respectively and then LTG treatment and PTZ stimulation were terminated for 1 week. After this washout period, PTZ stimulations continued in the absence of LTG once every other day 3 times to observe their behavior again. Thereafter, it was in vivo electrophysiological experiment followed with the histologic analysis.

### 2.7 Statistical analysis

Data were expressed as the Mean ± SEM. Two‐way ANOVA followed by Bonferroni posttest was used to analyze drug × time interaction on seizure stage. One‐way ANOVA followed by the Tukey Kramer Multiple Comparison post hoc test was used to analyze electrophysiological parameters, seizure stages and generalized seizure duration, and surviving neurons in different groups. Here *p *< .05 was considered statistically significant.

## 3 RESULTS

### 3.1 Effect of lamotrigine on anticonvulsant action

**Figure 1:**
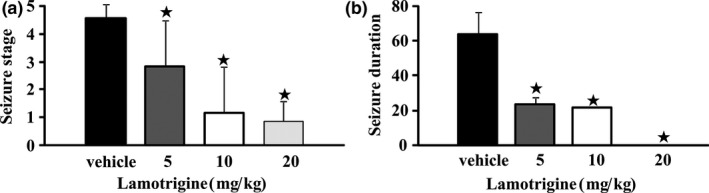
Effect of lamotrigine (LTG) on seizure stages (a) and seizure durations (b) in pentylenetetrazole (PTZ) kindled rats.Here 25 out of 30 rats were kindled after administration of PTZ (35 mg/kg, i.p.) once every other day for 15 times. Then different doses of LTG (5, 10, and 20 mg/kg; i.p.) or vehicle were treated 1 hour before PTZ (35 mg/kg) to test the anticonvulsant effect. The seizure stages and seizure durations were reduced significantly at different doses of LTG. Seizure duration was only measured when seizure stage at 4 or 5.The values are shown as Mean ± S.E.M. (*n *= 6–7), ^★^ *p* < .05 compared to the vehicle treatment group. Statistical analysis was performed by one way ANOVA followed by the Tukey Kramer Multiple Comparison post hoc test

After administration of PTZ (35 mg/kg) once every other day for 15 times, 25 out of 30 rats were kindled (seizure stages of 4 or 5 after three consecutive injections of PTZ).The kindled rats were randomly divided into 4 groups and the acute received LTG (5, 10 or 20 mg/kg) or vehicle for 1 hour before PTZ (35 mg/kg) to test the anticonvulsant effect. As shown in Figure 1, compared to the vehicle group with seizure stages and generalized seizure durations, significantly anticonvulsant effects were observed in the three LTG groups (*p* < .05). The mean seizure stages were 4.5 ± 0.5 in the vehicle group and 2.8 ± 1.6, 1.2 ± 1.6, and 0.9 ± 0.7, respectively, in the LTG (5, 10 or 20 mg/kg) groups. The mean generalized seizure durations were 64.3 ± 12.7 s, 24.0 ± 4.0 s, 22.0 ± 0.0 s, and 0 ± 0 s, respectively, in the vehicle and three LTG groups.

### 3.2 Effect of lamotrigine on seizure development

**Figure 2:**
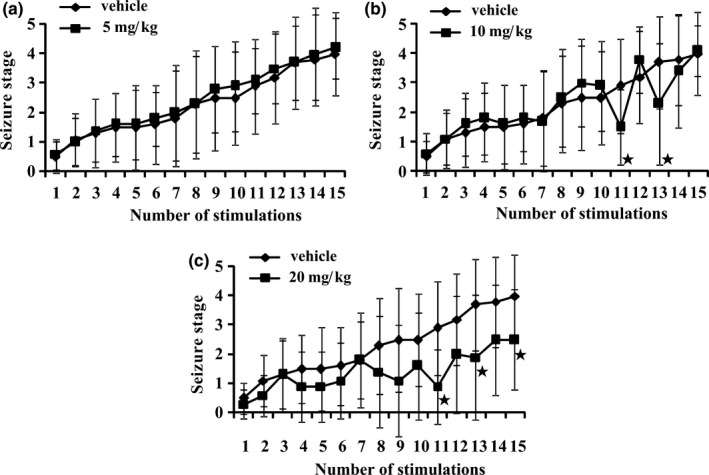
Effect of lamotrigine (LTG) treatment on seizure development in the kindling phase. Rats were stimulated with pentylenetetrazole (PTZ, 35 mg/kg, i.p.) once every other day for 15 times. One hour before each stimulation, animals received vehicle or different doses of LTG (5, 10, and 20 mg/kg; i.p.), respectively. The figure shows the mean seizure stages observed in each group during kindling development. (a) and (b) show 5 and 10 mg/kg LTG had no effect on seizure development. But a variable inhibition of the seizure stages by 10 mg/kg LTG was observed and some reached significance as indicated (^★^). (c) shows 20 mg/kg LTG inhibited the seizure development, for the seizure stages were reduced, especially at the 11th, 13th, and 15th stimulations. The values are shown as Mea*n *± SEM. (*n *= 10), ^★^ *p* < .05 compared to the vehicle treatment group. Statistical analysis was performed by two way repeat measures ANOVA followed by Bonferroni post test

Rats were stimulated by injections of PTZ (35 mg/kg) with LTG (5, 10 or 20 mg/kg) or vehicle 1 hour before once every other day for 15 times (*n *= 10). As shown in Figure 2a,b, during this kindling phase, compared to the vehicle‐treated group, treatment of LTG (5 and 10 mg/kg) did not influence the development of PTZ‐kindled seizures. However, LTG (10 mg/kg) inhibited seizure stages intermittently in the latter half of stimulations when most animals had generalized seizures (seizure stages of 4 or 5), i.e., stimulation 11 (seizure stage 1.5 ± 1.3 vs. 2.9 ± 1.6 for the 10 mg/kg LTG‐treated group vs. vehicle‐treated group, respectively; *p* < .05) and stimulation 13 (2.3 ± 2.1 vs. 3.7 ± 1.6, respectively; *p* < .05). As for the 20 mg/kg LTG‐treated group, the increases in seizure stage by PTZ stimulations were retarded significantly, for the animals remained longer in stage 1 to 3 than the vehicle‐treated group (Figure 2c, *p* < .05).

**Figure 3:**
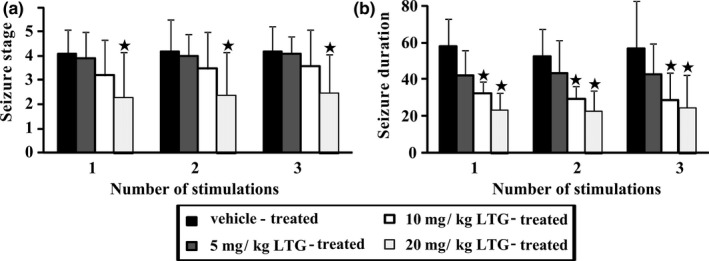
Effect of lamotrigine (LTG) treatment in the kindling phase on pentylenetetrazole (PTZ)‐induced seizure after a washout period. After the kindling process for 15 times, LTG (5, 10 and 20 mg/kg; i.p.) and PTZ (35 mg/kg, i.p.) were terminated for a week as the washout period. Then PTZ stimulations were carried out once every other day for 3 times again to test the effect of LTG on seizure development. (a) The rats previously treated with 20 mg/kg LTG showed a significantly lower in seizure stage compared with those previously treated with vehicle during the three stimulations. (b) The rats previously treated with 10 or 20 mg/kg LTG showed a significantly shorter in generalized seizure duration compared with those previously treated with vehicle too. The values are shown as Mean ± SEM (*n *= 8–10), ^★^ *p* < .05 compared to the vehicle‐treated group. Statistical analysis was performed by one way ANOVA followed by the Tukey Kramer Multiple Comparison post hoc test

Thereafter a washout period for a week without LTG treatments and PTZ stimulations, all survived rats (*n *= 8–10) were stimulated with PTZ again in the absence of LTG for 3 times. As shown in Figure 3a, the mean seizure stages in the 5 and 10 mg/kg LTG‐treated groups were not significantly different from those in the vehicle‐treated group, but for the 20 mg/kg LTG‐treated group they were significantly lower than those in the vehicle‐treated group (*p* < .05), which were 4.1 ± 1.0 vs. 2.3 ± 1.9 in stimulation 1, 4.2 ± 1.3 vs. 2.4 ± 1.8 in stimulation 2, and 4.2 ± 1.0 vs. 2.5 ± 1.6 in stimulation 3, respectively. As shown in Figure 3b, for the median generalized seizure durations, there was no significant difference between the vehicle‐treated group and 5 mg/kg LTG‐treated group too. However, the median generalized seizure durations in the 10 and 20 mg/kg LTG‐treated groups were obviously shorter than those in the vehicle‐treated group (*p* < .05), which were 32.8 ± 6.4 s, 23.8 ± 9.1 s and 58.3 ± 15.2 s, respectively, in stimulation 1, 29.9 ± 6.6 s, 23.3 ± 11.0 s and 52.8 ± 14.9 s, respectively, in stimulation 2, and 29.3 ± 14.4 s, 25.0 ± 17.6 s and 57.1 ± 25.9 s, respectively, in stimulation 3.

### 3.3 Electrophysiological experiments

**Figure 4:**
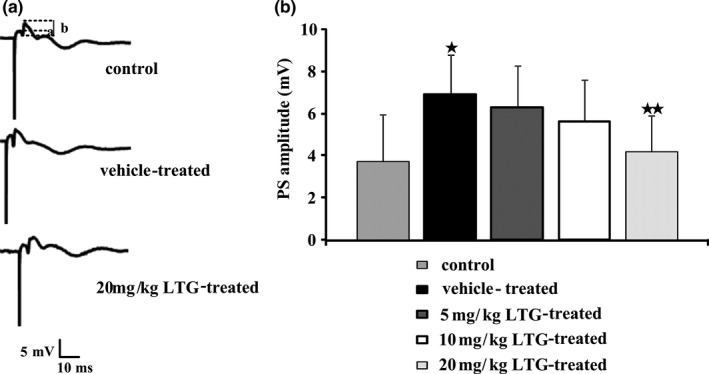
Population spike (PS) amplitude in the dentate gyrus as a function of perforant path at 0.1 mA stimulation current. Field potential were recorded in the hippocampus of anesthetized rats that were previously stimulated by pentylenetetrazole (PTZ, 35 mg/kg, i.p.) or vehicle with different doses of lamotrigine (LTG, 5, 10 and 20 mg/kg; i.p.) or vehicle. (a) Representative field potential in a control, a vehicle‐treated and a 20 mg/kg LTG‐treated rats. (b) Mea*n *± SEM values of PS amplitude in groups of 6–8 rats. PTZ stimulations increased the PS amplitude significantly, while 20 mg/kg LTG treatment previously afforded a reduction in the increased PS amplitude. ^★^ *p* < .05 compared to the control group, ^★★^ *p* < .05 compared to the vehicle‐treated group. Statistical analysis was performed by one way ANOVA followed by the Tukey Kramer Multiple Comparison post hoc test

After observing the behavior, the in vivo electrophysiological experiments were performed (*n *= 6–8). As shown in Figure 4b, increased amplitude of PS recorded in the dentate gyrus was a clear indication of neural hyperexcitability in the vehicle‐treated group (6.9 ± 1.9 mV vs. 3.7 ± 2.3 mV for the vehicle‐treated group vs. control group, respectively; *p* < .05). About 5 mg/kg and 10 mg/kg of LTG treatment for 15 times during the kindling phase did not change the increased PS amplitude (6.3 ± 2.0 mV and 5.6 ± 2.0 mV, respectively). However, the PS amplitude in the 20 mg/kg LTG‐treated group was significantly lower than in the vehicle‐treated group (4.2 ± 1.7 mV vs. 6.9 ± 1.9 mV for the 20 mg/kg LTG‐treated group vs. the vehicle‐treated group, respectively; *p* < .05).

**Figure 5:**
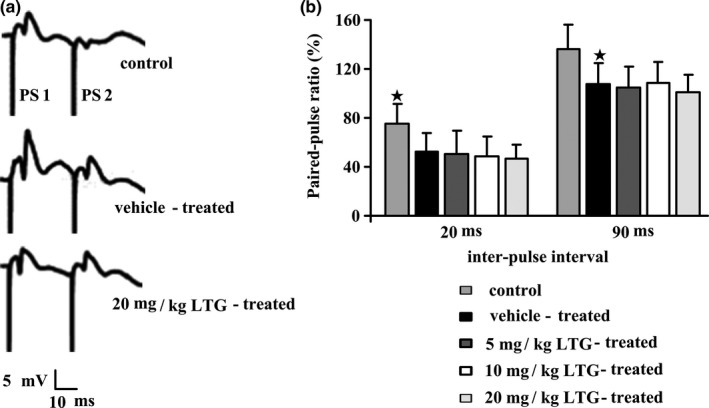
Paired‐pulse ratio of the population spike (PS) evoked in the dentate gyrus at interstimulus intervals 20 and 90 ms. Paired‐pulse inhibition or facilitation is quantified by the paired‐pulse ratio, defined as % amplitude of the PS2 vs. the amplitude of PS1. (a) Representative traces evoked with pairs of pulses at interstimulus interval 20 ms in a control, a vehicle‐treated and a 20 mg/kg lamotrigine (LTG)‐treated rats. (b) Mean ± SEM values of PS amplitude in groups of 6–8 rats. Pentylenetetrazole stimulations increased the paired‐pulse inhibition or decreased the paired‐pulse facilitation significantly, but different doses of LTG treatment did not change these increasing or decreasing. ^★^ *p* < .05 compared to the control group. Statistical analysis was performed by one way ANOVA followed by the Tukey Kramer Multiple Comparison post hoc test

Paired‐pulse indexes in the hippocampal DG area were strong in the control group at inter‐pulse intervals 20 and 90 ms. As shown in Figure 5b, PTZ kindling significantly increased paired‐pulse inhibition at inter‐pulse interval 20 ms in the vehicle‐treated group (52.0 ± 15.5% vs. 75.2 ± 15.9% for the vehicle‐treated group vs. control group, respectively; *p* < .05). However, 5, 10, and 20 mg/kg of LTG treatment for 15 times during the kindling phase did not change the increased paired‐pulse inhibition (50.8 ± 18.5%, 48.6 ± 15.9% and 46.6 ± 11.2%, respectively). Similarly, PTZ kindling significantly decreased paired‐pulse facilitation at the interpulse interval 90 ms in the vehicle‐treated group (107.4 ± 17.5% vs. 136.6 ± 19.8% for the vehicle‐treated group vs. control group, respectively; *p* < .05); and 5, 10, and 20 mg/kg of LTG treatment 15 times during the kindling procedure did not change the decreased paired‐pulse facilitation too, for they were 104.6 ± 17.2%, 109.0 ± 17.2%, and 101.4 ± 13.7%, respectively.

### 3.4 Histologic analysis

**Figure 6:**
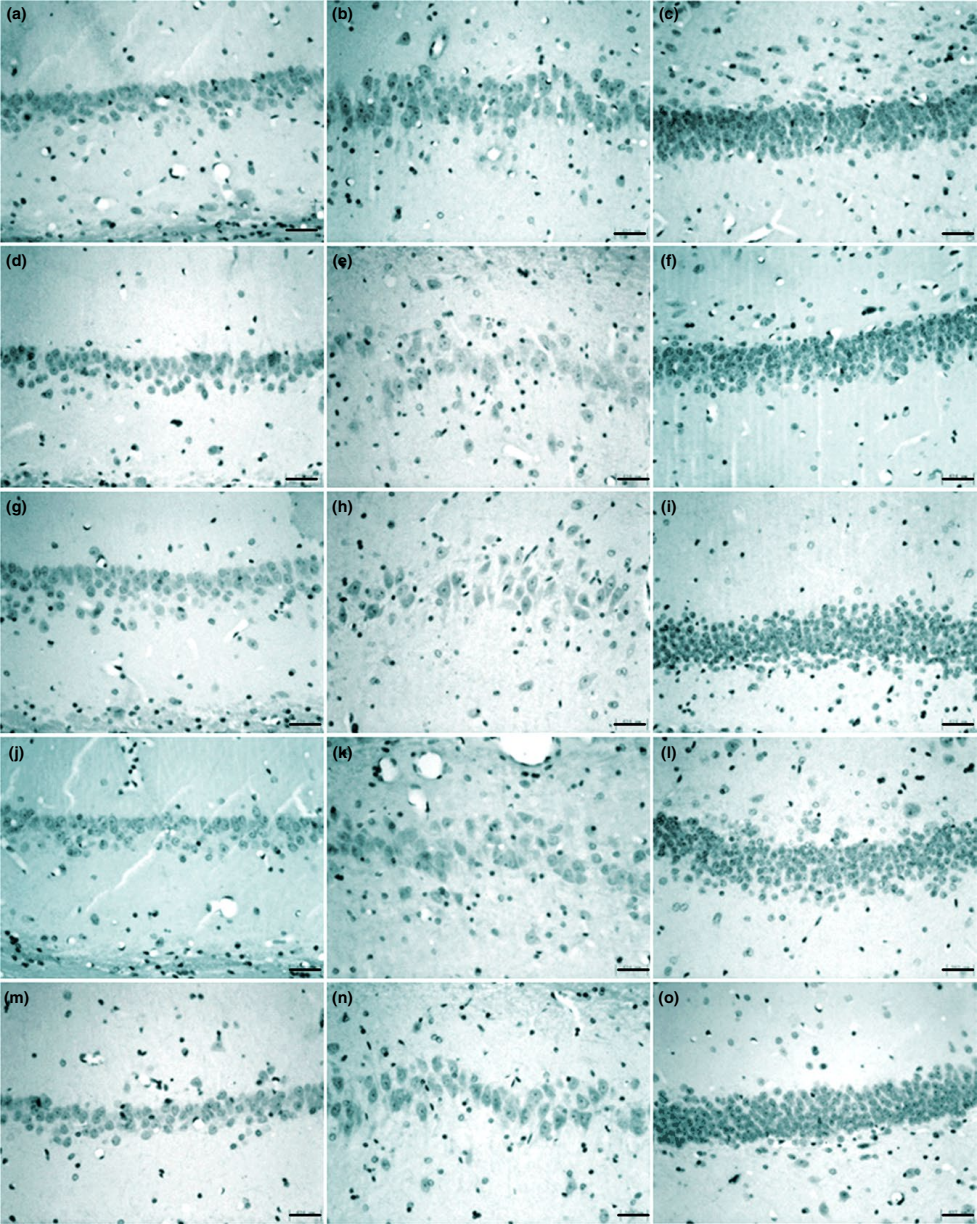
High power (200 × ) photomicrographs showing Nissl staining with cresyl violet of hippocampal neurons in different experimental groups. Normal cells showed round and pale stained nuclei. a–c show neurons in area CA1,CA3 and DG (dentate gyrus) in the control group from left to right, while d–f, g–i, j–l and m–o show neurons in corresponding areas in the vehicle‐treated and three lamotrigine (LTG)‐treated (5,10 and 20 mg/kg, respectively) groups. Data were obtained from 6–8 rats in each experiment group and the tatistical results are presented in Table 1. Scale bars: 400 μm

After electrophysiological experiments, the brains were removed, cut into sections, and stained with cresyl violet (*n *= 6–8). Normal cells showed round and pale‐stained nuclei. As showed in Figure 6 and Table 1, compared to the control group, there were no obvious neuron loss in the DG subfield in the vehicle‐treated and different LTG‐treated groups. However, the surviving neuron numbers significantly deceased in CA3 and CA1 subfields in the vehicle‐treated and three LTG‐treated groups (*p* < .05), but there were no obvious difference among the four groups.

**Table 1:** Effect of lamotrigine (LTG) treatments on neuron loss in different areas of hippocampus

	Surviving cells (1 mm length)		
	CA1	CA3	DG
Control	36.1 ± 7.6	51.8 ± 6.3	118.8 ± 10.4
Vehicle‐treated	22.4 ± 6.8^a^	16.8 ± 5.8^a^	106.5 ± 12.4
5 mg/kg LTG‐treated	23.8 ± 5.2^a^	17.3 ± 5.6^a^	102.7 ± 10.2
10 mg/kg LTG‐treated	24.5 ± 5.3^a^	19.0 ± 5.2^a^	105.4 ± 11.7
20 mg/kg LTG‐treated	26.2 ± 8.2^a^	20.6 ± 5.8^a^	108.6 ± 10.8

The number of surviving cells in CA1, CA3 and DG areas per 1 mm length in different groups was blindly counted under light microscopy. Compared to the control group, there were no obvious neuron loss in the DG subfield in the other four groups. There were no obvious differences in surviving cells in the CA3 and CA1 subfields in the four groups too, although the surviving cells in these subfields significantly deceased compared to the control one. Data are means ± SEM (*n *= 6–8).

a *p* < .05 vs. control group.

## 4 DISCUSSION

This study confirms that adequate doses of LTG can prevent seizure development in PTZ kindling rat model. After 20 mg/kg LTG pretreatment, the animals had seizures in stages varying from 1 to 3 for a long time in the kindling phase. Following cessation of LTG and PTZ for 1 week, and when PTZ stimulations were started, again the seizure stages in the 20 mg/kg LTG‐treated group were significantly lower than the vehicle‐treated rats; the generalized seizure durations in the 10 and 20 mg/kg LTG‐treated rats were significantly shorter too. Neuronal hyperexcitability can serve as a very sensitive marker of epilepsy (Margineanu, Matagne, Kaminski, & Klitgaard, 2008). Further, in vivo electrophysiological study also demonstrated 20 mg/kg of LTG obviously alleviated PTZ‐induced hyperexcitability, completely eliminating increased PS amplitude in the DG subfield of hippocampus. The half‐life of LTG in adult rat is 18–25 hr (Castel‐Branco, Falcao, Figueiredo, & Caramona, 2005; Yamashita, Furuno, Moriyama, Kawasaki, & Gomita, 1997), so a week washout period is enough for drug plasma level to decline below an effective level. Thus, the results have excluded the possibility that LTG simply masked the expression of kindled seizures through an anticonvulsant action. This indeed supports that LTG can inhibit the development of PTZ‐induced seizures in rats.

Kindling development is usually regarded as a process of epileptogenesis. Our study is the first reported demonstration of antiepileptogenic action by LTG in the PTZ kindling rat model. Previous studies for drug tolerance indirectly proved 5 mg/kg of LTG fails to inhibit the development of seizure in PTZ kindling rodents, paradoxically, leads to tolerance to a higher dose of LTG and carbamazepine (Singh et al., 2014; Srivastava et al., 2003). Similar results were found in the electrical kindling models for 5 mg/kg of LTG had no effect on the kindling development, which finally lead to tolerance of LTG and carbamazepine (Krupp et al., 2000; Postma et al., 2000). This phenomenon of tolerance usually occurs when administration of LTG before stimulations altered expression of voltage‐gated sodium channel subunits in brain tissue (Blumenfeld et al., 2009; Loscher, 2011; Srivastava, Alex, Wilcox, & White, 2013). However, there was a study that proved that a higher dose of LTG (20 mg/kg) can delay the development of seizures induced by electrical stimulations, although it cannot stop the final kindling acquisition (Stratton et al., 2003). In our study, 10 and 20 mg/kg of LTG can inhibit the development of seizure induced by PTZ too. Previous and present studies have demonstrated even 5 mg/kg of LTG has a marked a anticonvulsant effect in PTZ or electrical kindled rats, for it can produce a significant reduction in seizure stages, and seizure durations (Stratton et al., 2003). In adult Sprague‐Dawley rats, administration of 10 mg/kg of LTG results in a serum concentration of 4–5 μg/ml and 20 mg/kg in 7–8 μg/ml (Nissinen et al., 2004; Walton, Jaing, Hyun, & Treiman, 1996). In clinical monotherapy trials for newly diagnosed patients, LTG plasma concentrations are between 2 and 4 μg/ml (Brodie, Richens, & Yuen, 1995). Concentrations higher than 4 μg/ml, however, are usually used to control drug‐resistant epilepsy (Brodie et al., 1995). Therefore, a higher dose of LTG may be necessary to achieve the antiepileptogenic effect than the anticonvulsant action in the PTZ‐kindling rat model.

Some neuroprotective effects of LTG have been observed in cerebral ischemia, brain trauma, and post‐SE TLE models (Ketter et al., 2003; Li, Ketter, & Frye, 2002; Trojnar et al., 2002; Willmore, 2005). For example, previous studies found LTG can alleviate neuronal cell loss and mossy fiber sprouting in CA3 or CA1 regions of hippocampus in the post‐SE TLE animal models (Nissinen et al., 2004; Stables et al., 2003). Brain damage, including hippocampal neuronal cell loss, involving the epileptogenic process has long been widely accepted (Loscher & Brandt, 2010; Stepien, Tomaszewski, & Czuczwar, 2005; Trojnar et al., 2002). PTZ kindling does not require brain surgery or implantation of electrodes so the influence of brain trauma is ruled out. Unfortunately, the neuronal loss in hippocampus by PTZ stimulations did not get better by different doses of LTG treatments in our study. We did not observe mossy fiber sprouting and other neuropathological characteristic in our study that usually existed in the post‐SE TLE models because these pathological phenomenon are almost absent in the kindling model (Trojnar et al., 2002; Willmore, 2005). However, basic cellular changes in the process of kindling development can also be put as evidence by the macroscopic electrophysiological study (Gorter et al., 2016). In fact, our in vivo electrophysiological experiments proved 20 mg/kg of LTG significantly attenuates PTZ‐induced increasing in PS amplitude in the DG subfield of the hippocampus. Previous studies also found LTG significantly can decrease oxidative stress in pentylenetetrazole‐kindled animals (Agarwal, Agarwal, Mediratta, & Sharma, 2011; Arora et al., 2010). Thus, underlying the neuroprotective effect of LTG certainly exists in the PTZ kindling model in our study, which may be the pathological basis of preventing seizure development. More detailed pathological researches may be needed to determine the neuroprotective effect of LTG in the PTZ kindling rat model in the future.

The mechanism of antiepileptogenic action by LTG is uncertain. Recent researches proved phenobarbital and levetiracetam have the antiepileptogenic action in electrical kindling models, and they all have the potentiation of GABAergic inhibition (Loscher & Brandt, 2010; Radzik et al., 2015). Phenytoin and carbamazepine cannot suppress the development of kindling, and they are mainly blocking neuronal voltage‐gated sodium channels, and cannot increase the GABA‐mediated events (Loscher & Brandt, 2010; Radzik et al., 2015). Some other AEDs, such as LTG and valproate can block neuronal voltage‐gated sodium channels as well as increase the GABA‐mediated events, and both have antiepileptogenic actions in kindling model (Loscher & Brandt, 2010; Radzik et al., 2015). Therefore, it is speculated that the GABA‐mimetic effects could explain, at least in part, the antiepileptogenic activity of LTG in kindling model. This also reveals there may be some difference between antiepileptogenic and anticonvulsant targets. LTG is a drug with broad spectrum action targets, for instance, it can also block the calcium channels (N‐, P/Q‐, R‐, T‐type) and inhibit glutamate‐mediated events to also play the role of antiepileptogenic action through other ways (Messenheimer, 1995; Moeller et al., 2009; Morimoto et al., 2004). In fact, it was reported kindling‐induced long‐term potentiation in rat amygdala or hippocampus can significantly facilitate subsequent kindling, but LTG can inhibit the long‐term potentiation through blocking voltage‐dependent calcium channels (Gorter et al., 2016; Ketter et al., 2003; Morimoto et al., 2004). More detailed researches about the antiepileptogenic targets of LTG may be needed in the future.

Certainly, a cumulative effect of anticonvulsant action by LTG may also be involved in seizure development. Kindling is believed as a “seizures beget seizures” process as the number of stimulations increases, for with number of stimulations increasing amplification of seizure increases, finally culminating in generalized seizures (Hauser & Lee, 2002; Sills, 2007). During the initial kindling phase, the seizure degree is mild so that the anticonvulsant effect of LTG cannot be computed although it may really exist. In addition, the developed drug tolerance can mask the anticonvulsant role too. Finally the anticonvulsant effect accumulates and is falsely regarded as an antiepileptogenic effect. The drug tolerance phenomenon has been proved by previous studies, for example, rodents were treated with 5 mg/kg of LTG before each PTZ stimulation that led to a tolerance of 15 mg/kg of LTG finally, although this did not influence the seizure development (Singh et al., 2014; Srivastava et al., 2003). In our study, there was an increasing trend for seizure stage even in the 10 and 20 mg/kg LTG‐treated groups during the kindling phase, which indicates that the tolerance to LTG is developing because our study has proved that these doses of LTG can inhibit the seizure completely. One way of solving the problem is LTG was given after PTZ stimulation, but the long half‐life of LTG leads to unable to avoid the drug tolerance. Post‐SE models of TLE are also useful tools for antiepileptogenic treatment discovery (Stables et al., 2003). SE is a serious neurologic insult induced by a variety of chemical or electrical stimulations (Lowenstein, 1999). For post‐SE rodent models, SE is followed, after a latent period of days to weeks, by spontaneous recurrent seizures. The latent period offers an opportunity for antiepileptogenic therapies that can avoid the above problems. Unfortunately, previous studies proved LTG has no or determined antiepileptogenic effects in the post‐SE rodent models, which may be attributed to the shortage of experimental designs, for example, short sampling periods of video‐EEG monitoring (Margineanu et al., 2008; Stables et al., 2003). Recently, a study found just 4 PTZ injections followed by a noninjection window for nearly 3 weeks can induce a fully kindled state in rats (Davoudi et al., 2013). If it is proved to be a useful PTZ kindling model, administration of LTG in the noninjection window may be a choice to rule out the possible cumulative anticonvulsant effect of LTG.

## 5 CONCLUSIONS

This study supports that adequate doses of LTG treatment can prevent seizure development in the PTZ kindling rat model. More evidence may be needed to determine the effect of preventing is through only an antiepileptogenic action, or it with an anticonvulsant action of LTG. There are no data available about the antiepileptogenic effect of LTG in humans yet. Thus, whether administration of LTG could extend the therapeutic efficacy clinically deserves to be explored in future studies.

## CONFLICT OF INTEREST

None declared.
